# Effectiveness of Lymphatic Contrast Enhanced Ultrasound in the diagnosis of Cervical Lymph node metastasis from papillary thyroid carcinoma

**DOI:** 10.1038/s41598-021-04503-1

**Published:** 2022-01-12

**Authors:** Ying Wei, Yun Niu, Zhen-long Zhao, Xiao-jing Cao, Li-li Peng, Yan Li, Ming-an Yu

**Affiliations:** 1 Department of Interventional Medicine, China-Japan Friendship Hospital, No. 2 Ying-hua-yuan Street, Chao-yang District, Beijing, 100029 China; 2 Department of Pathology, China-Japan Friendship Hospital, No. 2 Ying-hua-yuan Street, Chao-yang District, Beijing, 100029 China

**Keywords:** Cancer, Head and neck cancer, Metastasis

## Abstract

Cervical lymph node metastasis (CLNM) is common in patients with papillary thyroid carcinoma (PTC), which is responsible for tumor staging and surgical strategy. The accurate preoperative identification of CLNM is essential. In this study, twenty consecutive patients with PTC received a parenchyma injection of Sonazoid followed by contrast enhanced ultrasound (CEUS) to identify CLNM. The specific lymphatic CEUS (LCEUS) signs for diagnosing CLNM were summarized, which were further compared with the resected specimens to get the pathological basis. After the injection of contrast agent, lymphatic vessel and lymph node (LN) could be exclusively displayed as hyperperfusion on LCEUS. The dynamic perfusion process of contrast agent in CLNM over time can be clearly visualized. Perfusion defect and interruption of bright ring were the two characteristic LCEUS signs in diagnosing CLNM. After comparing with pathology, perfusion defect was correlated to the metastatic foci in medulla and interruption of bright ring was correlated to the tumor seeding in marginal sinus (all p values < 0.001). The diagnostic efficacies of these two signs were high (perfusion defect vs. interruption of bright ring: AUC, 0.899, 95% CI 0.752–1.000 vs. 0.904, 0.803–1.000). LCEUS has advantages in identifying CLNM from PTC. The typical LCEUS signs of CLNM correlated with pathology.

## Introduction

Cervical lymph node metastasis (CLNM) often occurs in patients with papillary thyroid carcinoma (PTC), which is responsible for tumor staging and surgical strategy^1–4^. In cases with clinically metastatic lymph node (LN) involvement, central/lateral compartment neck dissection in addition to thyroidectomy is the standard procedure. However, in other cN0 low-risk cases, the role of prophylactic neck dissection remains open to debate^5^. Several authors cautioned against the systematic implementation of neck dissection because of surgical morbidity and the risk for hypoparathyroidism^6^. Furthermore, as the impact of micrometastasis on overall survival and local recurrence has not been clarified, the benefit of prophylactic surgery remains controversial in these cases^7^. Therefore, it’s very important to identify the CLNM preoperatively.

Routine ultrasound (US) is often used to diagnose CLNM^8, 9^. However, the typical signs of metastasis encountered in only a few cases, it is difficult to make definite diagnosis in most cases when there is small metastatic foci inside lymph node (LN)^9, 10^. Intravenous contrast enhanced ultrasound (IVCEUS), which is able to reveal tissue microvascularization, is used to identify CLNM recently^11^. However, it often does not work because it's difficult to identify the slight differences of enhancement between the minor metastatic foci and the surrounding lymphatic parenchyma in LNs attributed to the indolent feature of PTC. For example, the signs of homogeneous and iso-enhancement could be displayed both in benign and metastatic LNs^12^.

In recent years, several studies reported the use of lymphatic contrast enhanced ultrasound (LCEUS) to identify lymphatic vessels and sentinel lymph nodes in breast cancer and melanoma after peritumoral injections of the US contrast agent Sonazoid^13–15^. And a few preliminary studies focused on the application of LCEUS on cervical LNs. These results offered a few different ways in the diagnosis of CLNM^16, 17^. To date, there has been no report of applying LCEUS for the diagnosis of CLNM in PTC. Therefore, the purpose of present study is to summarize the characteristic LCEUS signs, as well as the pathological basis for the diagnosis of CLNM in PTC.

## Methods

### Patients

This prospective study design and protocol was approved by the Ethics Committee of China-Japan Friendship hospital (S2019-283-02). Written informed consent was obtained from each patient before the examination. The patients consented to the publishing of their anonymous examination results and radiological images. All methods were performed in accordance with the relevant guidelines and regulations.

Consecutive patients with PTC confirmed by pathology were enrolled. Patients with at least one of the following situations were excluded: (1) pediatric patients, (2) pregnant women, (3) patients who have a severe allergic reaction, (4) patients with a history of thyroidectomy, and (5) patients with tuberculous nodes or other metastatic cervical LNs confirmed by pathology.

All patients signed informed consent before examinations. All enrolled cases underwent LCEUS examination. The final diagnosis was confirmed by surgical pathology.

### LCEUS examination

A LOGIQ E9 US system (GE Healthcare, Waukesha, WI) equipped with a 6–15 MHz linear-array transducer was used. The mechanical index was 0.23 and the focus was located behind the target region. The contrast agent was Sonazoid (Daiichi-Sankyo, Tokyo, Japan). Any adverse events were recorded.

After the neck was sterilized, under the routine US guidance, 0.1 ml Sonazoid in 2 ml saline was injected with a 23-gauge needle into the superficial thyroid parenchyma in front of tumor. Ensure the contrast agent uniformly and completely dispersed around the tumor. In patients with more than one tumor, inject contrast agent in front of the largest tumor and observe the diffusion of contrast agent; if bilateral tumors, inject contrast agent on both sides. LCEUS was performed at different time points: 0–1, 5, 15, 30 min after the injection.

The observed contents included: the course of draining lymphatic vessels and the perfusion patterns of the target LNs. Perfusion pattern was further classified as: (1) complete perfusion—contrast agent fulfilled the entire LN, displayed as hyperenhancement, and (2) perfusion defect—absence of a filled area surrounding by a contrast-filled area within the LN. The bright ring refers to the circular enhancement along the edge of LN on LCEUS, which was further classified as: (1) complete bright ring—refers to unbroken circular enhancement along the edge of LN, and (2) interruption of bright ring—small perfusion defect in bright ring, which breaks the bright ring.

The results of LCEUS were analyzed by two radiologists (W.Y. and Y.M.A., with 5 and 15 year of experience in thyroid CEUS) who were blinded to the pathologic results. Because fine-needle aspiration (FNA) might cause atypia on pathology and cytology samples, FNA was not performed on LN to avoid affecting the outcome of LCEUS.

### Pathology examination

A contrast agent was injected into the thyroid parenchyma within 24 h before surgery to evaluate CLNM, and the target CLNM was labeled on the body surface. The patient was placed in a supine position with the neck hyperextended. Routine sterile skin preparation was performed. Under general anesthesia, thyroidectomy was performed by three surgeons with more than 5 years of clinical experience. The surgery strategies for total thyroidectomy and thyroid lobectomy followed the ATA guidelines^5^. A 5–8 cm transverse arc incision was made above the sterna notch, and the midline was opened. After the recurrent laryngeal nerve (RLN) and parathyroid gland were identified and protected, the thyroid gland was removed. According to the surface markers, target CLNM was firstly picking up and sent to pathological examination separately. Then, LN dissection was performed according to preoperative planning. Hemostasis was fully achieved, and the incision was sutured layer by layer.

Tissue samples were fixed in a 10% phosphate-buffered formalin solution, processed routinely and embedded in paraffin wax. Sections (4 μm) were stained with haematoxylin and eosin (H&E) to evaluate the histochemical properties of the tumor cells. Immunohistochemistry of cytokeratin (CK) and leukocyte common antigen (LCA) was further performed to specially mark tumor tissue and lymphatic structure.

The characteristic signs including the perfusion defect and interruption of bright ring on LCEUS were compared with the features of pathology in each LN. Pathologic diagnosis was evaluated by two pathologists (N.Y. and D.J.X, with 15 and 10 years of experience in PTC, respectively) who were blinded to LCEUS results.

### Statistical analysis

All statistical analyses were performed using SPSS software (version 20.0 for Windows; IBM, Armonk, NY). Receiver operating characteristic (ROC) curve analysis was used for the evaluation of sensitivity, specificity, accuracy, positive predictive value (PPV) and negative predictive value (NPV) of LCEUS. The overall ability of LCEUS in diagnosing CLNM was evaluated by calculating the area under the curve (AUC). Categorical variables were analyzed with a chi-square test, and continuous variables with independent t-test. P < 0.05 was considered to indicate significance.

## Results

### Baseline characteristics

From November 2019 to February 2020, a total of 34 LNs from 20 enrolled PTC patients (12 men, 8 women, mean age, 39.1 ± 11.3 years; range, 19–59 years) were evaluated. According to the pathological results, 26 were metastatic LNs and 8 were benign LNs. Twenty-two LNs were in lateral neck (16 metastatic and 6 benign LNs) and 12 were in center (10 metastatic and 2 benign LNs). The mean maximum diameters of the primary tumor and LNs were 1.0 ± 0.4 cm (range 0.3–1.7 cm) and 1.4 ± 1.0 cm (range 0.5–3.6 cm), respectively (Table 1). Compared with the pathological results, 9 LNs were diagnosed as benign on LCEUS, in which 7 is correct diagnosis, two is false negative diagnosis. Twenty-five LNs were diagnosed as CLNM on LCEUS, in which 24 is correct diagnosis, one is false positive diagnosis.

**Table 1:** Patient demographics and clinical characteristics.

Characteristic	Data
Patients	20
Gender (M/F)	12/8
Age (years)	39.1 ± 11.3
Primary tumor (n)	27
Maximum diameter (cm)	1.0 ± 0.4
Single (n)	14
Multiple (n)	6
Lymph nodules (n)	34
Maximum diameter (cm)	1.4 ± 1.0
Lateral neck (n)	22
Central neck (n)	12
Benign (n)	8
Malignant (n)	26

### Lymphatic vessels on LCEUS

**Figure 1:**
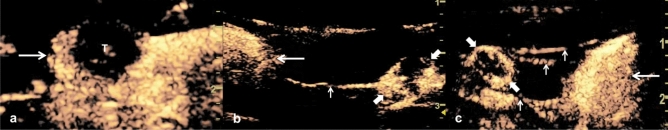
Lymphatic vessels on LCEUS. A 36-year male patient with PTC underwent LCEUS. (**a**) After subcapsular injection, the contrast agent slowly and uniformly diffuses in the thyroid parenchyma (long arrows), except for the primary tumor (T). (**b**) A lymphatic vessel (thin arrow) starts from the capsule of thyroid (long arrow), runs along the fascia and connects with one LN (thick arrows). (**c**) Three lymphatic vessels (thin arrows) connect with one LN (thick arrows). *PTC* papillary thyroid carcinoma, *LN* lymph node, *LCEUS* lymphatic contrast enhanced ultrasound.

After the administration of Sonazoid, the contrast agent gradually diffused in the thyroid parenchyma, except for the primary tumor (Fig. 1a). Typically, the contrast-enhanced lymphatic vessels starting from thyroid capsule could be observed within seconds. Lymphatic vessels displayed as a liner structure usually running along the capsule/fascia and connecting LNs (Fig. 1b). The enhanced lymphatic vessels could be clearly visualized in 17(17/22, 77.3%) lateral cervical LNs, but was absent in 12 central cervical LNs. The lymphatic drainage pattern—single LN with single lymphatic vessel was identified in 10 LNs, single LN with multiple lymphatic vessels was identified in 7 LNs (Fig. 1c).

### LCEUS vs. pathology in benign LNs

**Figure 2:**
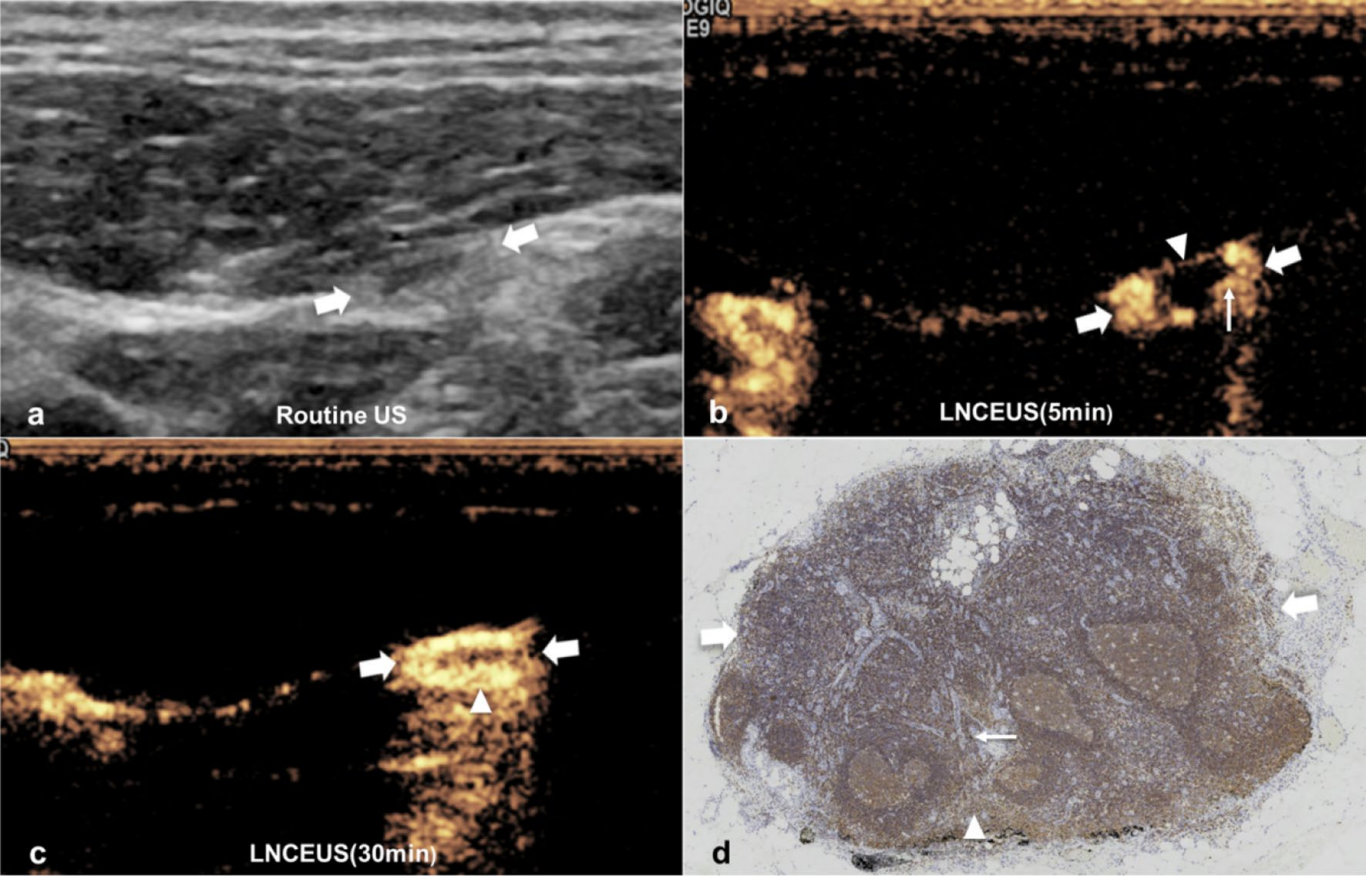
The dynamic perfusion process of contrast agent in benign LNs on LCEUS. A 28-year female patient with PTC underwent LCEUS. (**a**) Routine US shows a hypoechoic LN (thick arrows). (**b**) On LCEUS, the contrast agent first perfused into the edge of the LN (thick arrows), presenting as a bright ring (arrowhead), and then diffused centripetally (thin arrow). (**c**) The contrast agent almost completely diffused in the entire LN (thick arrows), displaying as homogenous perfusion. (**d**) Normal marginal sinus (arrowhead) and peritrabecular sinus (thin arrow) within LN (thick arrows) were showed on LCA staining (× 50), which matched the complete bright ring and the centripetal enhancement area inside LN on LCEUS, respectively. *PTC* papillary thyroid carcinoma, *LN* lymph node, *LCEUS* lymphatic contrast enhanced ultrasound, *LCA* leukocyte common antigen.

The perfusion pattern of benign LNs is centripetal. Firstly, complete bright rings were displayed in the sub-capsule of the LNs, just matched the areas of marginal sinus on anatomy. Subsequently, the contrast agent centripetally perfused to the center of LN, just correlated to the areas of pertrabecular sinus on anatomy. Finally, the contrast agent completely diffused in the entire LNs, which displayed as homogenous enhancement (Fig. 2). The dynamic perfusion process of contrast agent in LNs over time can be clearly visualized. In benign LNs, contrast agent perfused homogeneously and completely. According to the pathology including H&E, CK and LCA, those LNs had normal structure and no metastatic foci were found inside them. Of the 8 benign LNs, 7 (7/8, 87.5%) were correctly diagnosed by LCEUS. One was misdiagnosed as CLNM, and pathology confirmed that ‘perfusion defect’ might be a region of peritrabecular sinus where the contrast agent didn't fulfill in observing time.

### LCEUS vs. pathology in CLNM

According to the study on the different signs of LCEUS, perfusion defect and interruption of bright ring were the two specific signs for the diagnosis of CLNM. The sensitivity and specificity of both signs (perfusion defect vs. interruption of bright ring) were 92.3% vs.80.8% and 87.5% vs. 100% (Table 2). Two CLNMs had been misdiagnosed as benign LNs because the micrometastatic foci with maximum diameter less than 1.5 mm on pathology had not been disclosed by LCEUS.

**Table 2:** Diagnostic efficacies of perfusion defect, interruption of bright ring and both of them in CLNM.

LCEUS signs	AUC (95% CI)	Sensitivity (%)	Specificity (%)	PPV (%)	NPV (%)	Accuracy (%)
Perfusion defect	0.899 (0.752–1.000)	92.3	87.5	96.0	77.8	91.2
Interruption of bright ring	0.904 (0.803–1.000)	80.8	100	100	61.5	85.2
Both signs	0.962 (0.898–1.000)	92.3	100	100	80.0	94.1

*LCEUS* lymphatic contrast enhanced ultrasound, *AUC* area under curve, *PPV* positive predictive value, *NPV* negative predictive val.

**Figure 3:**
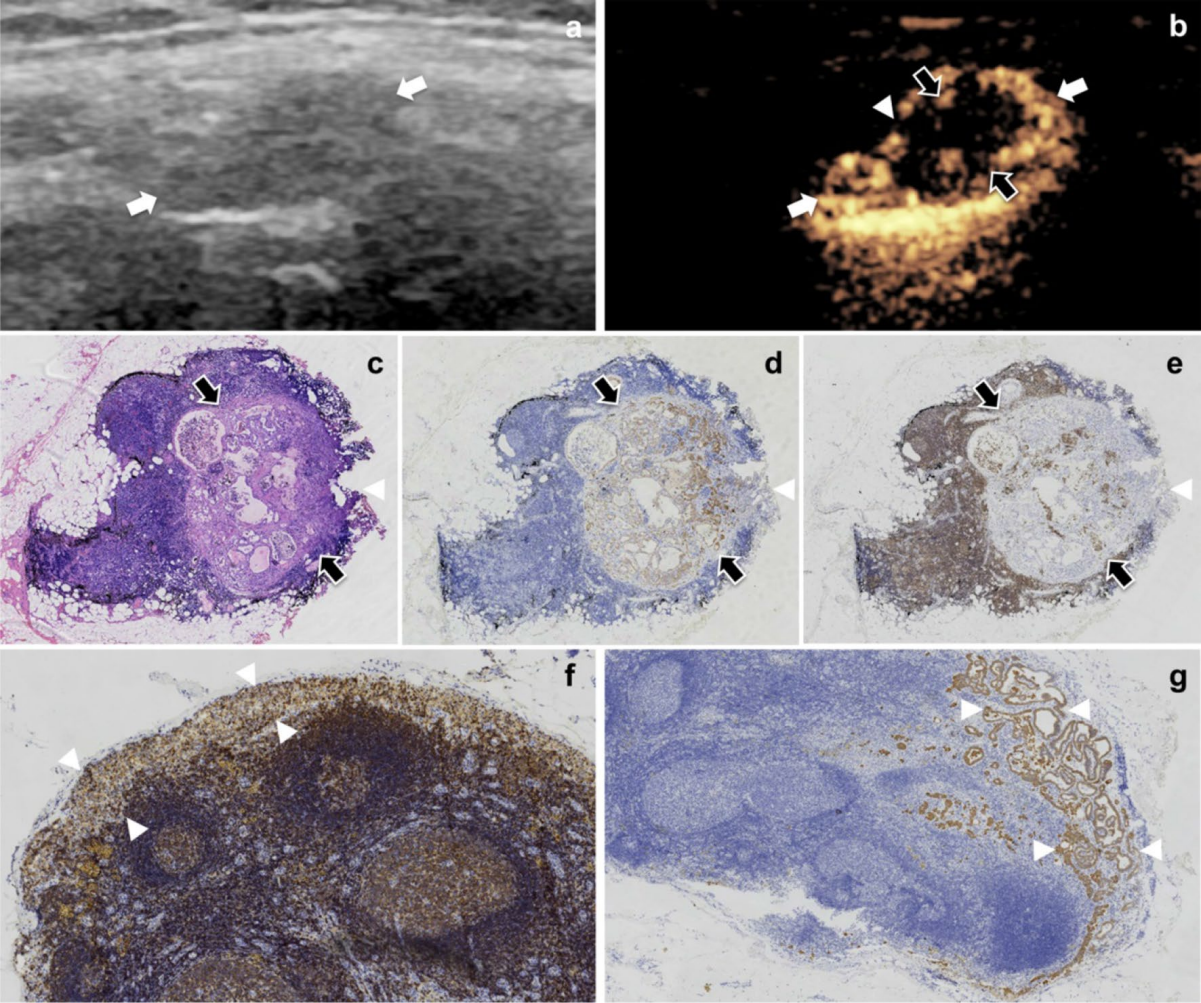
The characteristic of metastatic foci on routine US, LCEUS and pathology. A 46-year male patient with PTC underwent LCEUS. (**a**) The metastatic foci in a LN (white arrows) could not be identified on routine US. (**b**) The metastatic foci (black arrows) in LN (white arrows) were clearly visualized as perfusion defect (black arrows) and interrupted bright ring (arrowhead) on LCEUS. (**c**) Photomicrograph of surgically resected specimens shows metastatic foci (black arrows) invasion in medulla and marginal sinus (arrowhead) (H&E, × 20). (**d**) The metastatic foci (black arrows) within medulla and marginal sinus (arrowhead) were specially marked through CK staining (× 20). (**e**) Lymphatic tissue was specially marked by LCA staining while the metastatic foci (black arrows) were negative for LCA staining (× 20), which corealated to perfusion defect and interrupted bright ring on LCEUS. (**f**) Normal marginal sinus (arrowheads) was showed on LCA staining (× 50). (**g**) Marginal sinus (arrowheads) damaged by tumor invasion on CK staining, matching the interruption of bright ring on LCEUS (× 50). *LN* lymph node, *H&E* hematoxylin–eosin, *LCA* leukocyte common antigen, *CK* cytokeratin, *LCEUS* lymphatic contrast enhanced ultrasound, *US* ultrasound.

A comparing study was performed between the LCEUS signs and the tumor in LNs. The result showed both signs including perfusion defect and interruption of bright ring were highly correlating to the tumor inside LNs on pathology (all p value < 0.001) (Table 3). For one LN, the perfusion defect on LCEUS just correlated to the tumor in medulla on pathological section, regarding the size, number and location. Generally, the tumor could be identified on H&E staining. Furthermore, the CK staining specially marked the tumor cells, which made sure that the tumor exclusively localized at the position where the perfusion defect emerged on LCEUS. LCA was employed to specially mark the lymphatic structure, which further made sure there was rich lymphatic drainage around the tumor, but was almost absent inside the tumor. Similarly, the interruption of bright ring just correlated to the small foci inside the marginal sinus on pathology including H&E, CK and LCA (Fig. 3).

**Table 3:** Comparison between LCEUS signs and pathological changes.

	LCEUS signs	Pathological changes	p
	Perfusion defect	Metastatic foci	
Present	25	26	<0.001
Absent	9	8	

	Interruption of bright ring	Interruption of marginal sinus	
Present	21	26	<0.001
Absent	13	8	

*LCEUS* lymphatic contrast enhanced ultrasound.

**Figure 4:**
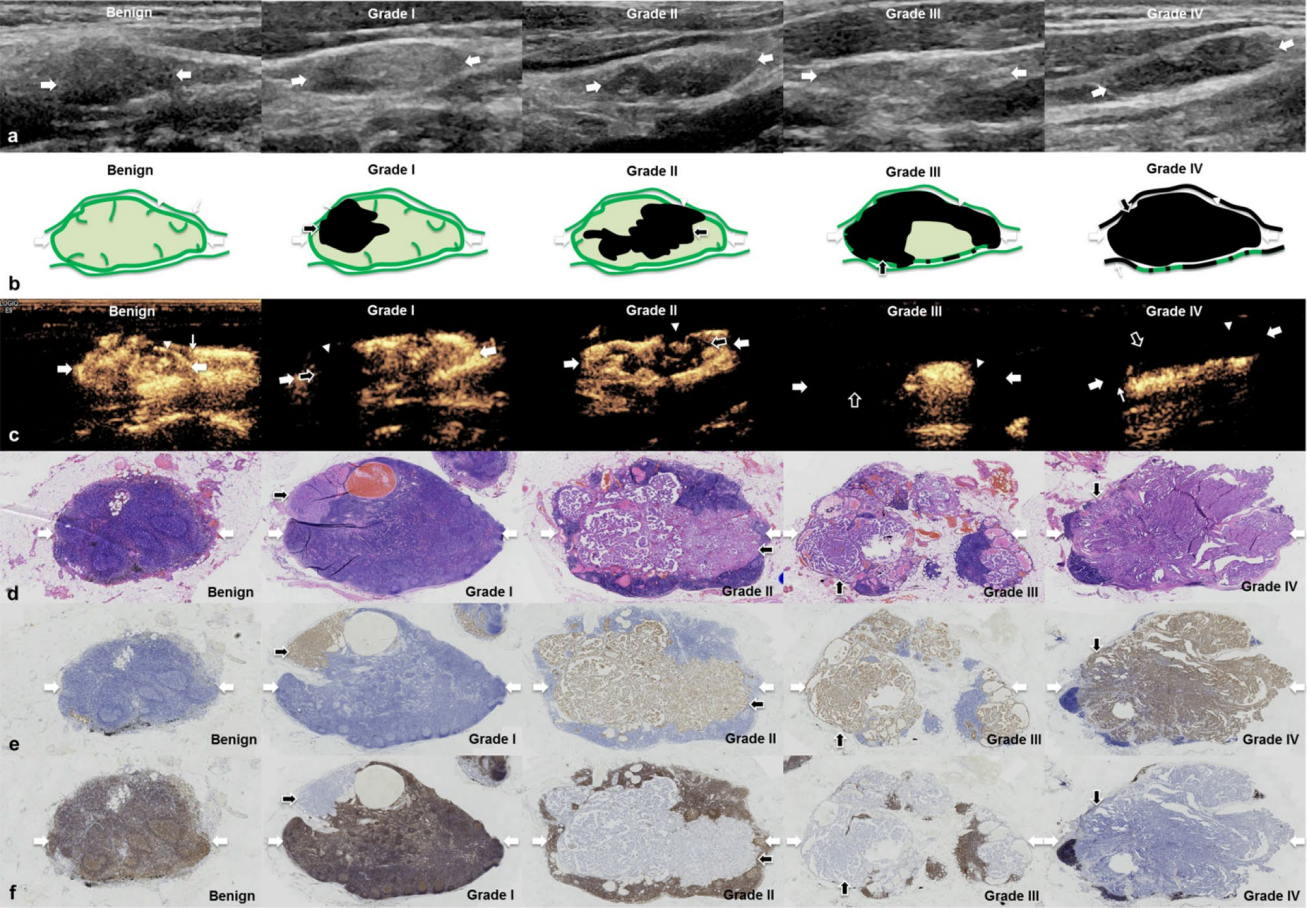
Different grades of CLNM on routine US, LCEUS and pathology. (**a**) Benign LNs and different grades of CLNM (thick arrows) on routine US. (**b**) Schematic of metastatic foci (black arrows)growing in parenchyma, marginal sinus (arrowheads), and lymphatic vessels (thin arrows) in different grades of LNs (thick arrows). (**c**) LCEUS shows perfusion defects (black arrows) developing within parenchyma, marginal sinus (arrowheads) and lymphatic vessels in different grades of LNs (thick arrows). The interruption was found in bright ring (arrowheads in Grade I to Grade IV) and lymphatic vessel (thin arrow in Grade IV). (**d**) Photomicrograph of surgically resected specimens shows tumor growing (black arrows) (H&E, × 10) (**e**) The dynamic process of tumor growth was showed on CK staining (CK, × 10). With the growth of tumor, the small foci (black arrows from Grade I to Grade IV) enlarged, fused with each other and occupied the entire LNs. (**f**) The growing of tumor (black arrows) leaded to progressive loss of lymphatic structure within LNs (thick arrows) (LCA, × 10). *LN* lymph node, *CLNM* cervical lymph node metastasis, *US* ultrasound, *LCEUS* lymphatic contrast enhanced ultrasound, *CK* cytokeratin, *LCA* leukocyte common antigen.

According to the proportion of the perfusion defect area/the entire LN area on the maximum section and the continuity of lymphatic vessels, the status of LN metastasis was further classified as four grades: grade I (≤ 30% perfusion defect), grade II (30–60% perfusion defect), grade III (60–90% perfusion defect) and grade IV (≥ 90% perfusion defect + lymphatic vessel interruption (Fig. 4)). Four (4/24, 16.7%) CLNMs were classified as grade I, 5 (5/24, 20.8%) as grade II, 6 (6/24, 25.0%) as grade III and 9 (9/24, 37.5%) as grade IV. Of the 15 CLNMs classified as grades I to III, 5 had one perfusion defect on LCEUS, and 10 had two or more perfusion defects. The median diameter of the perfusion defect was 9 mm, ranged from 2.0 to 32 mm.

### Adverse events

One patient experienced erythema around injection site. The symptoms were relieved after antallergic and asymptomatic treatment.

## Discussion

As an obvious risk indicator of poor prognosis, CLNM occurs in approximately 35–80% of patients with PTC^2, 5, 18–21^. The accurate preoperative identification of CLNM could trigger the lateral dissection. Similarly, the definite diagnosis of benign LN could prevent unnecessary LN dissection, and reduce the risks of complication^22, 23^. Routine US is generally applied to assess CLNM preoperatively, however, the value is controversial due to its inconsistent sensitivity that ranges from 33 to 70%^24^. In last years, IVCEUS was used to differentiate the microvascular features of metastases from those of normal tissue. However, the ability of IVCEUS to differentiate the minor differences of enhancement between small metastatic foci and the surrounding lymph parenchyma is limited^11, 12^.

According to the results of the present study, LCEUS not only successfully displayed the process of lymphatic drainage, but also clearly disclosed the LN architecture, which was highly consistent with the pathological findings in detail. In benign LNs, the dynamic centripetal perfusion process was clearly visualized. More importantly, two specific signs—perfusion defect and interruption of bright ring, which had promising values in diagnosing CLNM, were proposed. In fact, both signs mostly reflected the small tumor within LN through the comparison with the pathology. The LCEUS sign of perfusion defect correlated to metastatic foci in medulla. The interruption of bright ring correlated to the tumor seeding in the marginal sinus. Furthermore, LCEUS not only has high tissue resolution on the diagnosis of CLNM, but also has high spatial resolution, which could identify the tumor with maximum diameter as small as 2 mm inside LN. Certainly, the LN with too small metastatic foci sometimes could not be displayed by LCEUS. Conversely, perfusion defect on normal structure could be misdiagnosed as metastasis in case of insufficient perfusion time.

By classifying the LCEUS images of CLNM and comparing them with pathological specimens, further findings were obtained in current study. The dynamic process of tumor growth in LNs could be analyzed as follows: first, small metastases seed in the medulla and/or marginal sinus of LN. In those areas, the lymphatic fluid drainage is very slow, which could provide a suitable microenvironment for tumor growth. Even if the metastasis is small about 2 mm, it could be captured by LCEUS. Second, the growth and fusion of micrometastases lead to a large perfusion defect on LCEUS. As a result, the normal lymphatic parenchyma is compressed. Third, when the metastases occupy the entire LN, there is usually no perfusion in the input lymphatic vessels, which indicates the forming of tumor emboli, resulting in distant metastases^25^. A high correlation between LCEUS findings and pathological changes fully demonstrates the advantages of LCEUS in diagnosing CLNM.

According to the results in present study, the advantages of LCEUS are summarized as follows: (1) the lymphatic drainage is relatively slow, so the moving of microbubbles within lymphatic vessels and LNs could be easily visualized by LCEUS in real time, which indicated a high temporal resolution. (2) After the injection of contrast agent into thyroid parenchyma, the lymphatic system exclusively shows hyperperfusion, suggesting the higher tissue resolution of LCEUS. (3) In the present study, the LCEUS not only showed the slender lymphatic vessels but also disclosed the minor metastatic foci with maximum diameter of larger than 2 mm, which indicated a high spatial resolution of LCEUS. (4) The injection of contrast agent into the thyroid parenchyma can simulate the physiological process of lymphatic drainage, which leads to a more objective and complete demonstration of lymph drainage, and (5) the comparatively long persistence time of microbubbles in vivo, guaranteeing the full evaluation of all suspected LNs^13–15^.

Up to now, there are only a few preliminary studies focused on the identification of metastatic LNs by LCEUS^16, 17^. Comparing with the present study, where the generation and flow of lymphatic fluid, as well as the process of perfusion inside the lymphatic vessels and LN could be clearly and objectively disclosed on LCEUS through direct injection of contrast agent into thyroid parenchyma, and two typical signs on diagnosis of CLNM were summarized and further compared with pathology, there are several deficiencies in previous researches. Regarding directly injection of contrast agent into the sub-capsular region of LN to distinguish benign and metastatic LNs, it is troublesome to fully evaluate multiple suspected LNs in one procedure^16^. And the administration route was not conforming to the natural circulation process of lymphatic drainage, which made it difficult to guarantee the objectivity and completeness of assessment. In another study^26^, although it is favorable for describe the thyroidal lymphatic drainage, that research was merely carried out in an animal experiment with a very few sample. There are a few limitations in the present study. First, the sample size in the present study is relative small, and the next further studies with large samples might help to provide more definite results. Second, the pathology of lymphatic vessels was not obtained; the mechanism of metastasis relating lymphatic vessel, as well as the role of LCEUS on disclosing the rule should be made clear in further study. Third, there was no comparison between LCEUS results and CT scans. Fourth, the subtypes of PTC have not been classified, and further studies will be improved based on pathological subtypes.

## Conclusion

LCEUS has advantages in diagnosing CLNM from PTC. The LCEUS signs of CLNM correlate well with pathology.
